# Pontine tegmental cap dysplasia accompanied by a duplicated internal auditory canal

**DOI:** 10.1590/0100-3984.2016.0015

**Published:** 2017

**Authors:** Rodolfo Mendes Queiroz, Lara Zupelli Lauar, Luiz Carlos Alves de Souza, Rafael Gouvêa Gomes de Oliveira, Lucas Giansante Abud

**Affiliations:** 1 MED - Medicina Diagnóstica / Hospital São Lucas, Ribeirão Preto, SP, Brazil.; 2 Clínica Paparella de Otorrinolaringologia, Ribeirão Preto, SP, Brazil.

**Figure 1:**
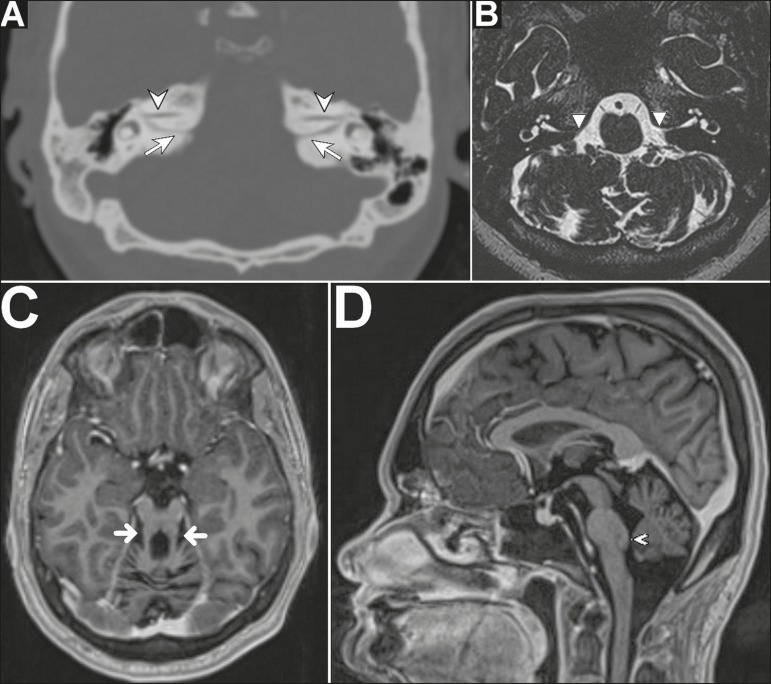
**A:** Oblique axial CT scan of the ears, with bone window settings, showing a narrow, duplicated internal auditory channel, one channel containing the facial nerve (arrowhead) and the other containing the vestibulocochlear nerve (arrow). **B:** Axial T2-weighted MRI scan of the brain and ears, revealing the absence of the eighth cranial nerve (arrowhead). **C:** Oblique axial T1-weighted, volumetric, intravenous contrast-enhanced MRI of the brain and ears, showing elongated, discretely lateralized superior cerebellar peduncles (arrows), similar in appearance to the molar tooth sign. **D:** Sagittal T1-weighted, volumetric, intravenous contrast-enhanced MRI of the brain and ears, showing cerebellar hypoplasia with a dysplastic aspect and with a reduction in the volume of the pons, especially in its ventral aspect, presenting a small prominence on the posterior surface projecting into the fourth ventricle (arrowhead).

Dear Editor,

A 48-year-old female with cognitive and auditory deficits presented for evaluation prior to cochlear implantation. Among her parents and four siblings, there was one brother with mental disability of unknown cause. Physical examination revealed ataxia. An electrophysiological study of hearing revealed the absence of waves from the cochlear nerve and of auditory brain-stem pathways evoked by 95 dB nHL clicks and 500-1000 Hz tone bursts (also at an intensity of 95 dB nHL). There was also an absence of otoacoustic emissions in both ears, indicating profound sensorineural hearing loss. Computed tomography (CT) of the ears showed a narrow, duplicated internal auditory canal, one canal containing the facial nerve and the other containing the vestibulocochlear nerve (Figure 1A), together with a discrete reduction in the volume of the pons and cerebellum. In addition to the duplicated internal auditory canal, magnetic resonance imaging (MRI) of the brain and ears revealed the following: absence of the eighth cranial nerve (Figure 1B); elongated, discretely lateralized superior cerebellar peduncles, with an appearance similar to the molar tooth sign (Figure 1C); pons with a dysplastic aspect and a reduction in its volume, especially in the ventral region, presenting a small prominence, on the posterior surface, projecting into the fourth ventricle; and cerebellar hypoplasia, mainly in the vermis (Figure 1D). On the basis of those findings, the patient was diagnosed with pontine tegmental cap dysplasia (PTCD).

Cerebellar hypoplasia/hypogenesis can be seen in cases of metabolic disorder, exposure to teratogens, congenital infection or genetic disorders^(1)^. The molar tooth sign is observed in the axial plane of CT scans and, more clearly. of MRI scans at the junction between the rhombencephalon and mesencephalon, classically in the presence of cerebellar vermis hypoplasia/agenesis, deep interpeduncular fossa; Superior, poorly oriented, thickened and elongated superior cerebellar peduncles^(2)^.

PTCD is a brainstem malformation^(1,3,4)^, initially described in 2007 by Barth et al.^(5)^; to date, fewer than 50 cases have been reported^(4)^. The main signs and symptoms are auditory deficiency, in 92% of cases; cognitive deficit, in 76%; deglutition disorders, in 64%; facial paralysis, in 60%; abnormal eye movement, in 60%; trigeminal paresthesia, in 60%; ataxia, in 56%; hypotonia; cyclic vomiting syndrome; and various neurological disorders of the third to the eighth cranial nerves^(4,6,7)^. Other potential characteristics of PTCD include hypoplasia of the pons (notably in its ventral aspect); a mass of ectopic dorsal pontine fibers protruding into the fourth ventricle; hypoplasia/agenesis of the middle and inferior cerebellar peduncles; elongation of the superior cerebellar peduncles; cerebellar vermis hypoplasia/ agenesis; absence or malformation of the inferior olivary nuclei; hypogenesis/absence of the third to eighth cranial nerves; costovertebral deformities; and cardiovascular anomalies^(1,3-8)^. PTCD can exhibit a feature similar to the molar tooth sign, although with lateralized, tapered superior cerebellar peduncles^(1,4,6)^. The differential diagnoses include pontocerebellar hypoplasia, as well as a number of syndromes^(2,8,9)^: Joubert; Dekaban-Arima; Senior-Loken; COACH; Váradi-Papp; Malta; and Moebius.

Although a duplicated internal auditory canal is extremely rare, it is found in at least 46% of all cases of PTCD. The two canals are often narrow (with a caliber of less than 2.0 mm) and accompanied by hypogenesis/agenesis of the eighth cranial nerve, which typically contraindicates cochlear implantation^(4,8,10,11)^. Some authors have reported differentiated cases in which the division is made by a bony septum, proposing that the term "partitioned" (rather than "duplicated") be used in such cases^(11)^.
